# Selective recovery of silver by thiuram disulfide-modified cellulose

**DOI:** 10.1039/d5ra09818a

**Published:** 2026-02-24

**Authors:** Shunsuke Taka, Keisuke Nakakubo, Yuma Ito, Tsuyoshi Taniguchi, Masaru Endo, Kuo H. Wong, Asami S. Mashio, Tatsuya Nishimura, Katsuhiro Maeda, Hiroshi Hasegawa

**Affiliations:** a Graduate School of Natural Science and Technology, Kanazawa University Kakuma Kanazawa Ishikawa 920-1192 Japan; b Integrated Research Center for Circular Technology, National Institute of Advanced Industrial Science and Technology (AIST) 16-1 Onogawa Tsukuba Ibaraki 305-8569 Japan k.nakakubo@aist.go.jp +81 50-3522-5393; c Catalytic Chemistry Research Institute, National Institute of Advanced Industrial Science and Technology (AIST) 1-1-1 Higashi Tsukuba 305-8565 Ibaraki Japan; d Daicel Corporation 1239 Shinzaike, Aboshi-ku Himeji-Shi Hyogo 671-1283 Japan; e Institute of Science and Engineering, Kanazawa University Kakuma Kanazawa Ishikawa 920-1192 Japan hhiroshi@se.kanazawa-u.ac.jp +81 76-234-4792; f Nano Life Science Institute (WPI-NanoLSI), Kanazawa University Kakuma Kanazawa Ishikawa 920-1192 Japan

## Abstract

A thiuram disulfide–modified cellulose adsorbent (TDMC) was developed for the highly selective recovery of silver ions from aqueous solutions. The adsorbent was synthesized by introducing thiuram disulfide functional groups onto a cellulose backbone to selectively adsorb Ag(i). Batch adsorption experiments were conducted to evaluate the adsorption performance and selectivity under various conditions. The maximum adsorption capacity of TDMC for Ag(i), estimated from the Langmuir model, was 3.61 mmol g^−1^ at pH 1.0. Remarkably, TDMC exhibited outstanding selectivity toward Ag(i) even in the presence of high concentrations of competing metal ions. When Ag(i) coexisted with Cu(ii), Pb(ii), Zn(ii), Ni(ii), Ca(ii), K(i), Mg(ii), and Na(i) at 2000-fold higher total concentrations, the adsorption percentage of Ag(i) remained 99.0%, comparable to that under single-metal conditions, whereas those of other ions were below 2.5%. X-ray photoelectron spectroscopy revealed that adsorbed Ag atoms were bonded to sulfur atoms of the thiuram disulfide groups. Furthermore, fast atom bombardment mass spectrometry was performed on a low-molecular-weight thiuram–Ag(i) complex that exhibits spectroscopic features similar to those of Ag–TDMC, allowing us to determine the molecular weight and structural characteristics of the complex. These findings demonstrate that TDMC possesses an exceptional ability to selectively capture Ag(i) through specific Ag–S coordination, providing a simple and efficient approach for silver recovery.

## Abstract 2

Thiuram-disulfide-grafted cellulose enables highly selective Ag(i) adsorption even in the presence of Pb(ii) and high concentrations of competing ions.

## Graphical abstract

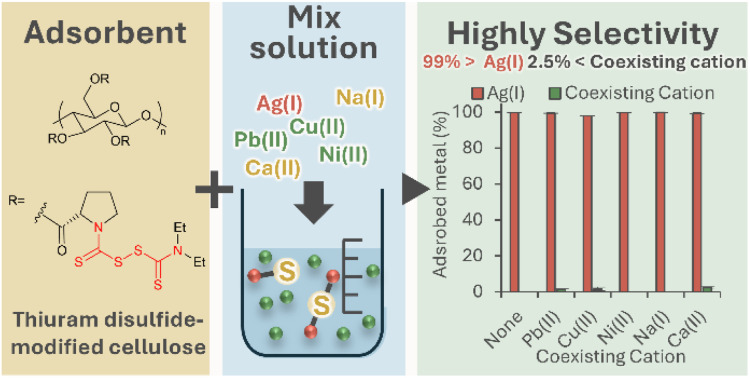

## 1. Introduction

Silver (Ag) is widely used not only in jewelry but also in diverse fields, including electronics, photography, and medicine.^1,2^ In recent years, Ag has also been utilized as an electrode material in crystalline silicon-based solar cells, and its continued demand is anticipated.^3,4^ However, silver is a limited resource, and its demand has been increasing. Accordingly, the recovery of silver from secondary resources such as electronic substrates and solar panels has garnered increasing attention. In addition to resource concerns, Ag(i) is known to pose risks to human health and the environment. For instance, the accumulation of Ag(i) in the human body can cause argyria, which is a condition characterized by blue-gray discoloration of the skin.^5^ Therefore, the recovery of silver from secondary resources and the removal of Ag(i) from the environment are paramount.

Ag(i) can be recovered using various methods, among which adsorption is widely used because of its operational simplicity and rapid processing.^6^ Various adsorbents have been investigated, and examples used in practical processes include activated carbon and chelating resins.^7,8^ Although activated carbon has been applied to the recovery of Ag(i) from cyanide leachates, it generally exhibits low selectivity and typically, requires additional purification or selective leaching to obtain high-purity silver.^9,10^ This limitation has motivated the development of chelating resins with enhanced selectivity.

Various functional groups, such as macrocyclic ligands containing sulfur and oxygen atoms,^11^ oxygen-rich macrocycles,^12^ sulfydryl groups,^13^ thiourea groups^14^ and polyhydroxy-capped poly(amidoamine) functionalities,^15^have been introduced to enhance selectivity. When macrocyclic compounds are used, metal ions can be separated through host–guest interactions based on ion recognition. However, if the host–guest interaction serves as the basis for selectivity, then the separation of ions with similar ionic radii becomes challenging. For instance, Ag^+^ (1.00 Å) and Pb^2+^ (0.98 Å) possess almost nearly identical ionic radii, thus rendering their separation difficult.^16,17^ Other adsorbents exhibit disadvantages such as low selectivity, which renders separation from Cu(ii) and Pb(ii) challenging^13,14^ or results in low adsorption capacity for Ag(i).^15^

Previously, we developed a dithiocarbamate-modified adsorbent that exhibited high adsorption capacities for precious metals such as Au(iii) and Ag(i).^18,19^ However, this adsorbent co-adsorbed base metal ions such as Pb^2+^ and Cu^2+^, and their removal *via* acid washing introduces an additional purification step that increases process complexity and operational costs.^18^ Such lows electivity is a critical limitation for practical applications, particularly in the recovery of high-purity silver from complex secondary resources.

Additionally, the abovementioned adsorbent captures Ag(i) by forming coordination bonds through orbital interactions with the sulfur atom, whereas competing ions such as Pb^2+^ and Cu^2+^ are speculated to be adsorbed through electrostatic interactions with the negatively charged sulfur atoms of dithiocarbamate ligands. Therefore, neutralizing the electrical charge of the functional groups may potentially suppress the undesired adsorption of base metal ions without sacrificing the intrinsic affinity toward Ag(i).

Based on this concept, we focused on the dimerization of dithiocarbamate groups, which neutralizes the negative charge and yields a neutral thiuram disulfide. Thiuram disulfides can be synthesized easily, and their S–S bond and electron distribution are expected to alter the adsorption behavior toward metal ions. To the best of our knowledge, no adsorbent bearing a dimerized structure—namely, the thiuram disulfide group—has been reported, nor has its application to precious-metal recovery been investigated despite its potential advantages in selectivity control.

In this study, we investigate the adsorption capacity, selectivity, and adsorption mechanism of Ag(i) onto a thiuram disulfide-modified cellulose (TDMC) adsorbent and evaluate its potential as a high-performance material for the selective recovery of Ag(i).

## 2. Materials and methods

### 2.1. Chemical reagents

Silver nitrate (Kanto Chemical, Tokyo, Japan) was used to prepare a stock solution. Nitrate salts of Na(i), K(i), Ca(ii), Mg(ii), Ni(ii), Cu(ii), Zn(ii), Pb(ii) as well as standard solutions of Ag(i) and previous eight elements (1000 mg L^−1^) and thiourea were purchased from Kanto Chemical. Acetic acid (Kanto Chemical), sodium acetate (Nacalai Tesque, Kyoto, Japan), 4-(2-hydroxyethyl)-1-piperizineethanesulfonic (HEPES) were used as buffers for pH 3.0, 5.0, and 7.0. Sodium hydroxide and nitric acid were used to adjust the pH of solution.

### 2.2. Instrumentation

ICP-OES (iCAO6300 or iCAP PRO XP, Thermo Fisher Scientific, MA, USA) was used to determine the metal concentration in the solution. FT-IR analysis was performed using an FT-IR-6800 spectrophotometer (JASCO, Tokyo, Japan) in the wavenumber range of 500–4000 cm^−1^, using the KBr pellet method. XPS analysis was conducted using an FPS-9010 spectrometer (JASCO, Tokyo). The XPS sample were pressed onto indium foils for fixation and subsequently placed on a sample holder with carbon tape. XPS measurements were performed using a Mg Kα X-ray source under conditions of 10 kV, 10 mA, 5.0 × 10^−6^ Pa. Fast–atom–bombardment Mass Spectroscopy (FAB-MS) was conducted using JMS–700 in the mass-to-charge ratio (*m*/*z*) of 50–900, with nitro benzyl alcohol or glycerol as the matrix.

### 2.3. Synthesis of adsorbent

A thiuram disulfide-modified cellulose (TDMC) was synthesized from the previously reported adsorbent.^20^ To a solution of sodium *N,N*-diethyldithiocarbamate (24.7 g, 109.8 mmol) in water (24 mL) was added dropwise a 30% solution of H_2_O_2_ (11 mL, 109.8 mmol) at 0 °C, and the resultant solution was stirred for 50 min at the same temperature. After a dithiocarbamate-modified cellulose adsorbent (DMC) synthesized according to our previously reported study (3.3 g, 3.66 mmol) was added to the solution, the resultant mixture was immediately acidified below pH 1 with conc. H_2_SO_4_ at 0 °C. Subsequently, MeOH (59 mL) was added to this mixture, followed by stirring for 4 h at room temperature. The formed precipitate was collected, washed with MeOH and Et_2_O, and dried *in vacuo* to give a TD-modified cellulose adsorbent (TDMC) (2.56 g, 62% yield) as pale-yellow solids. Afterall, TDMC was ground using a mortar and pestle to a particle size of less than 212 µm. The synthesis protocol is illustrated in Fig. S1.

### 2.4. Batch adsorption protocol

The adsorbent (2.5 mg) and 5.0 mL of metal solutions were added to 50 mL centrifuge tubes. The tubes were then shaken for 1.0 or 3.0 h at 200 rpm and 25 ± 0.2 °C. The metal concentrations were varied depending on the purpose of experiments (100–6000 µM). After agitation, the mixtures were filtrated by 0.45 µm filters, and the metal concentrations of the initial solutions and filtrates were quantified *via* ICP-OES. Additionally, the amount of metal adsorption (mmol g^−1^) and percent of metal adsorption were calculated using the following equation:

1 Adsorbed metal (mmol g^−1^) = (*C*_i_ − *C*_e_)*V*/*m*

2 Adsorbed metal (%) = (*C*_i_ − *C*_e_)/*C*_i_

where *C*_i_ and *C*_e_ represent the initial and equilibrium concentrations of metals (mM), *V* refers to the volume of the metal solution (L), and *m* corresponds to the weight of the adsorbent (g).

### 2.5. Desorption protocol

A Ag(i)-loaded adsorbent (2.5 mg) and 5.0 mL of eluents were placed into 50 mL centrifuge tubes and agitated for 1.0 h at 200 rpm and 25 ± 0.2 °C. After desorption, the metal concentrations in the solutions before and after the process were quantified using ICP-OES. Additionally, the desorption percentage was calculated using eqn (3):3
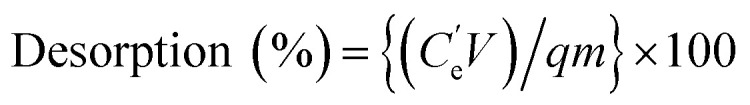
where 
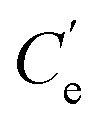
 and *V* represent the concentration of desorbed Ag(i) (µM) and the eluent volume (L), respectively, and *q* and *m* correspond to the adsorbed Ag(i) (µmol g^−1^) and the Ag(i)-loaded adsorbent used (g), respectively.

## 3. Results and discussion

### 3.1. Characterization of TDMC

**Fig. 1:**
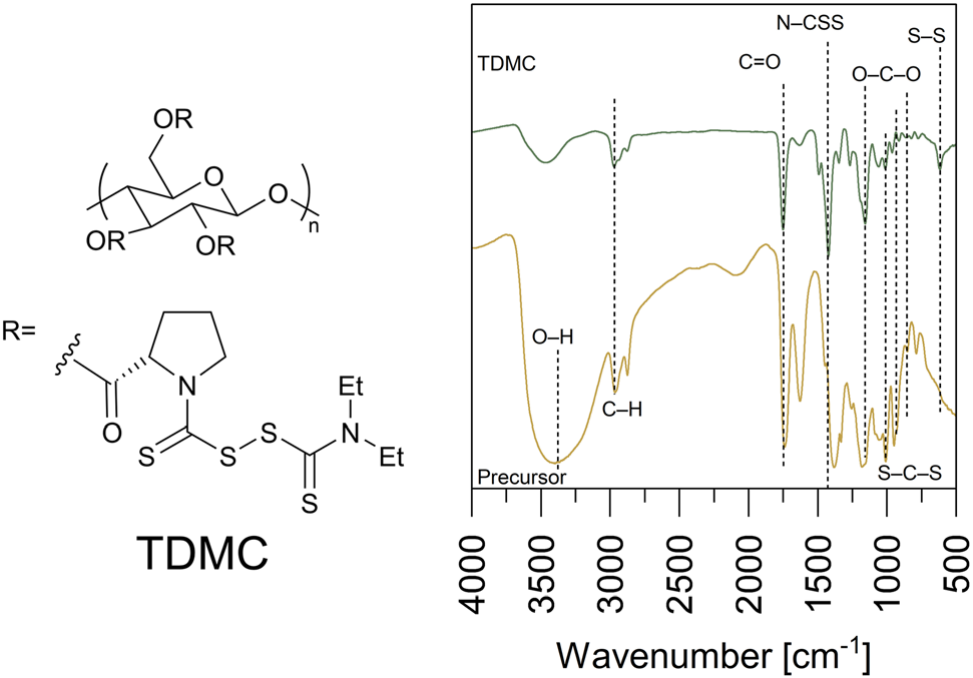
Chemical structure of TDMC and FT-IR spectra of the precursor and TDMC.

For TDMC (Fig. 1), the bands at 3461 and 2971 cm^−1^ are assigned to O–H and C–H stretching.^20^ The intense peaks at 1750, 1422, and 1157 cm^−1^ correspond to C=O, N–CSS, and S–C–S, respectively.^20,21^ Additionally, a new sharp band at 615 cm^−1^, which is assigned to S–S stretching, was observed in TDMC. This band was absent in the precursor, thus supporting the formation of a thiuram disulfide structure *via* the dimerization of the dithiocarbamate groups.^22^

### 3.2. Effects of pH and solution acidity

**Fig. 2:**
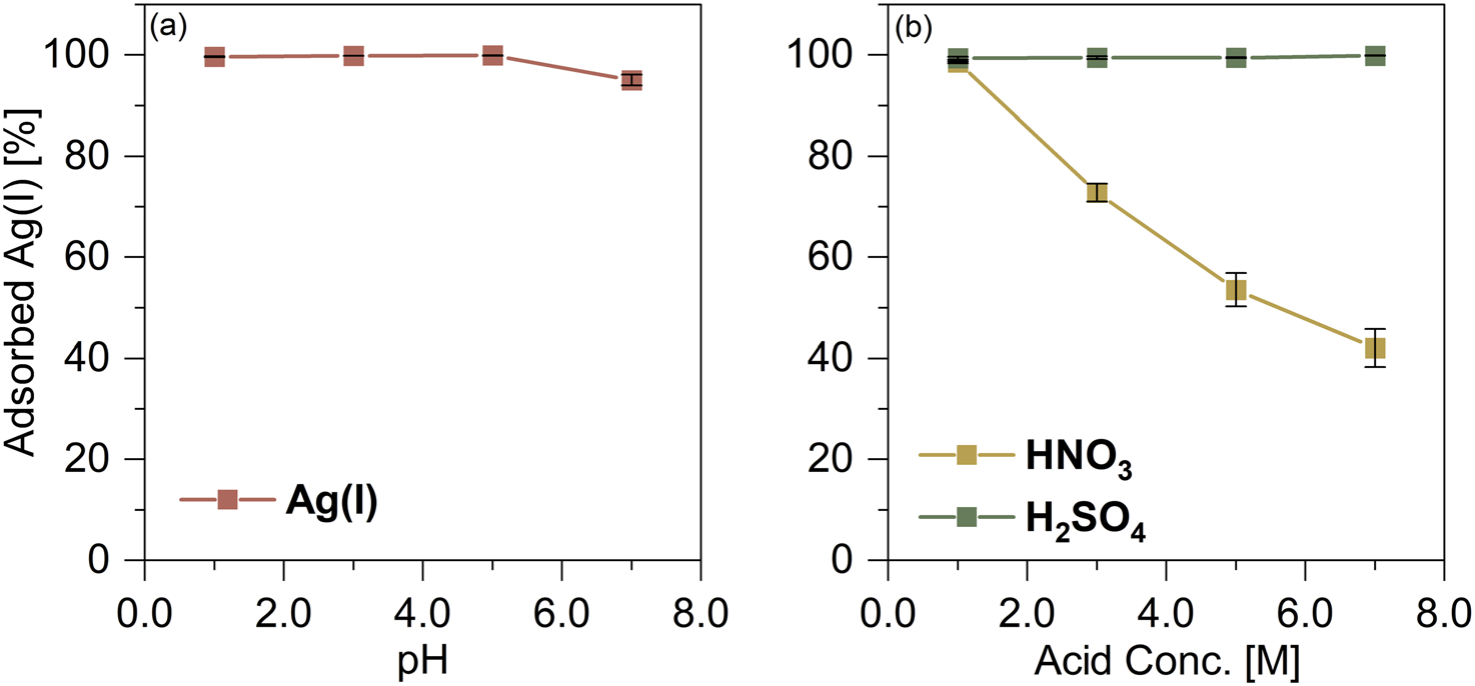
Effects of (a) pH and (b) acids on Ag(i) adsorption. Ag(i) conc. = 500 µM, contact time = 1.0 h, dose = 5.0 mg.

pH and solution acidity can affect the speciation of adsorbates, thus resulting in diverse adsorption behavior. The effects of pH and solution acidity on Ag(i) adsorption by TDMC were investigated. The percentages of adsorbed Ag(i) were >99.5% at pH 1.0–5.0. Meanwhile, the percentage of Ag(i) adsorbed decreased to 95.0% at pH 7.0 (Fig. 2a). Although HEPES which has a N/O ligand has long been regarded as a non-coordinating buffer, it has been shown to form a CuL^+^ complex with Cu(ii) (log *β* = 3.22), thus demonstrating measurable metal-binding ability.^23^ Moreover, N/O-donor ligands have been reported to form complexes with Ag(i).^24,25^ By analogy, the N/O donor sites in HEPES may weakly coordinate to Ag(i). As HEPES is typically used at relatively high concentrations (10–100 mM), even weak complexation can reduce the ratio of free Ag^+^ in solution. Therefore, we consider HEPES as a plausible inhibitory factor for Ag(i) adsorption at pH 7.0, which is consistent with the decreased uptake observed (Fig. 2a).

The effect of solution acidity on Ag(i) adsorption depends on the acid types shown in Fig. 2b. At any tested concentrations of H_2_SO_4_, the percentage of Ag(i) adsorbed consistently exceeded 99.0%. However, under HNO_3_ conditions, the adsorbed Ag(i) gradually decreased from 98.6% to 42.0% as the HNO_3_ concentration increased from 1.0 to 7.0 M. This pronounced decrease is attributable to the oxidation of sulfur atoms in TDMC *via* the strong oxidization of HNO_3_, whereas H_2_SO_4_ does not exhibit such oxidizing behavior. Sulfur donors such as thioethers and disulfides are generally classified as soft bases—according to HSAB theory—and thus exhibit high coordination affinity toward Ag(i)—a soft acid. However, oxidation to sulfoxides or sulfones converts these donors into harder ones, thus significantly decreasing their affinity for Ag(i).^26–28^ As discussed in the mechanism section, TDMC captures Ag(i) through coordination to sulfur atoms; therefore, the oxidation of these sites provides a consistent explanation for the reduced adsorption observed in HNO_3_.

### 3.3. Adsorption isotherm

**Fig. 3:**
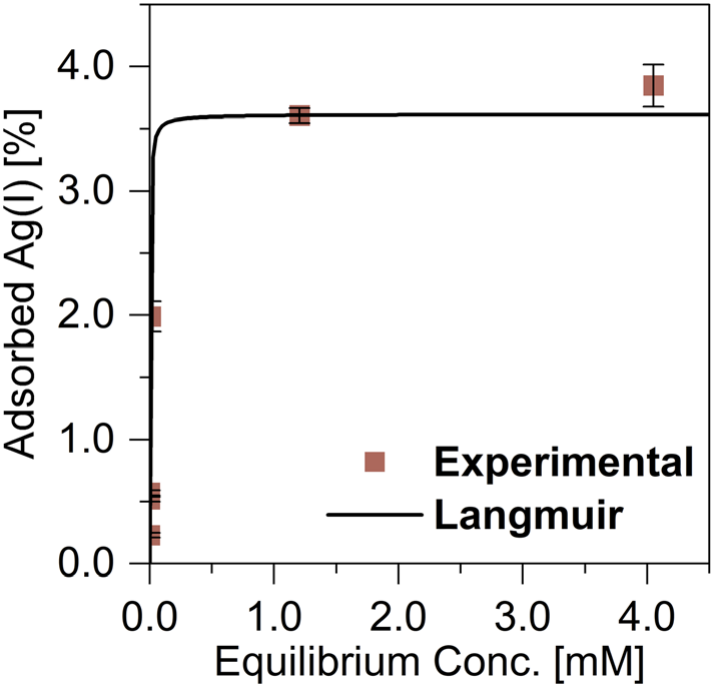
Effects of initial Ag(i) concentration on Ag(i) adsorption. pH = 1.0, contact time 1.0 h, dose = 5.0 mg.

The initial concentrations of an adsorbate affect the quantities of its adsorption. The effect of the initial Ag(i) concentration on its adsorption was examined at pH 1.0 to determine the maximum monolayer adsorption capacity. The equilibrium data were fitted using the Langmuir and Freundlich isotherm models (Fig. 3), and the obtained parameters and correlation coefficients are summarized in Table 1^29,30^ The Langmuir model provided an excellent fit (*R*^2^ = 1.000), and surpassed the Freundlich model (*R*^2^ = 0.920). The maximum monolayer adsorption capacity of TDMC for Ag(i) was calculated to be 3.61 mmol g^−1^.

**Table 1:** Constants obtained from the Freundlich and Langmuir isotherms

Freundlich parameters			Langmuir parameters		
*K*_F_ [(mmol)^(1 − 1/*n*)^ g^−1^ L^−1/*n*^]	*n*	*R* ^2^	*Q* [mmol g^−1^]	*K*_L_ [L mmol^−1^]	*R* ^2^
4.28	4.03	0.920	3.61	387	1.000

### 3.4 Effects of coexisting ions

**Fig. 4:**
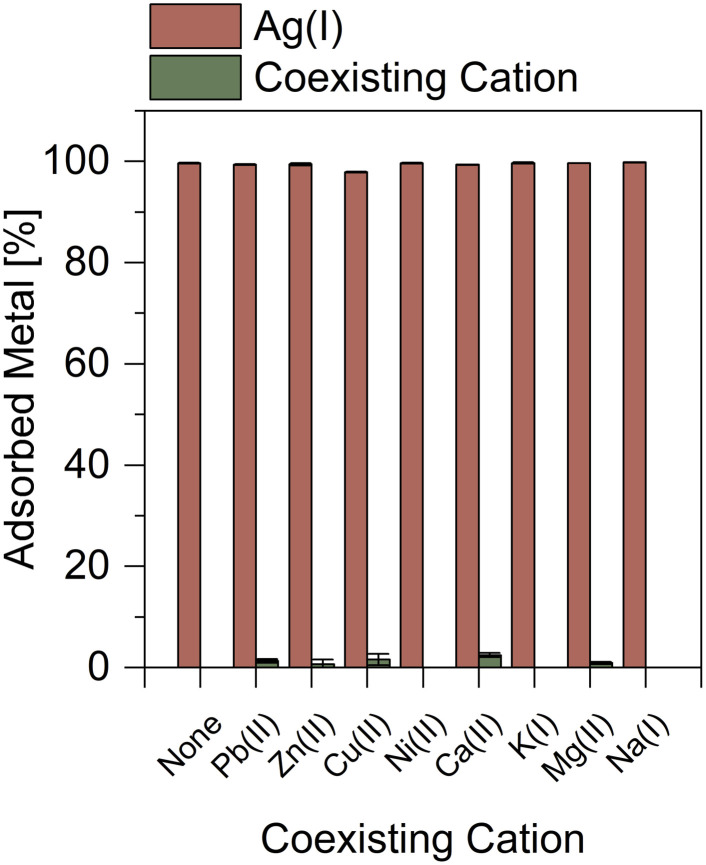
Effects of coexisting ions on Ag(i) adsorption. pH = 1.0, Ag(i) conc. = 500 µM, metal conc. = 1.0 M, contact time = 1.0 h, dose = 5.0 mg.

To evaluate the selectivity of TDMC, the adsorption of Ag(i) was examined in the presence of various coexisting cations, including Cu(ii), Pb(ii), Zn(ii), Ni(ii), Ca(ii), K(i), Mg(ii), and Na(i) (Fig. 4). Even when the concentrations of these metal ions were 1.0 M, which is 2000 times higher than that of Ag(i), TDMC adsorbed Ag(i) almost quantitatively without any noticeable interference. The adsorption percentages of the coexisting ions were less than 2.5%, thereby demonstrating the exceptional selectivity of TDMC toward Ag(i). Although TDMC does not surpass previously reported adsorbents in terms of adsorption capacity, its selectivity is substantially superior (Table 2). TMMR and G0-MT possess multiple binding sites, including S donors as well as abundant N-containing functionalities (*e.g.*, thiourea nitrogen and/or amines). Because these N sites can be protonated and participate in electrostatic/ion-exchange–like interactions, the overall uptake is not governed solely by soft Ag–S coordination; consequently, these materials can capture various heavy-metal ions under competitive conditions, thereby complication strict Ag selectivity. Meanwhile, ASPSS4 is bifunctional (thiol + amine): whereas thiols favor Ag(i), the additional amine sites broaden the range of possible interactions and can reduce “Ag-only” selectivity in multi-ion systems. A/M-CDMOF-gel is designed for the simultaneous adsorption of multiple ions (*e.g.*, Au^3+^/Ag^+^/Pb^2+^) and thus is not Ag-selective by design. Notably, TDMC avoids the adsorption of Cu(ii) and Pb(ii), which is a significant disadvantage of DMC, further highlighting its improved performance for selective Ag(i) recovery.

**Table 2:** Comparison of maximum adsorption capacity and metal ions co-adsorbed between TDMC and reported cellulose-based adsorbents

Adsorbent	*Q* [mmol g^−1^]	pH/acidity temperature (°C)	Metal ions co-adsorbed	Reference
TMMR	5.58	5.0, 25	Al(iii), Mg(ii), Fe(iii), Cd(ii), Pb(ii)	32
G0-MT	1.10	6.0, 25	Cu(ii)	33
ASPSS4	4.43	6.0, 35	Cu(ii)	31
A/M-CDMOF-gel	0.56	6, —	Pb(ii)	34
DMC	10.97	0.2 M HNO_3_, 55	Cu(ii), Pb(ii)	19
TDMC	3.61	1.0, 25	None	This work

This high selectivity can be rationalized based on the chemical nature of TDMC. Dithiocarbamates are anionic *S*,*S*-donor ligands that form complexes with metal ions through both electrostatic and soft–soft orbital interactions.^35^ By comparison, the sulfur donor atoms in TDMC are electrically neutral, which reduces the relative contribution of electrostatic interactions in metal–ligand binding. Under such conditions, the neutral sulfur donors can exhibit low affinity toward divalent metal ions such as Pb(ii), Cu(ii), and Zn(ii) which are borderline acids under HSAB theory and therefore exhibit significantly reduced adsorption under our conditions.^28^ By contrast, the neutral soft sulfur donors in TDMC bond with Ag(i) —a soft acid—and forms strong Ag–S bonds, thus, its adsorption persists even when electrostatic effects are reduced.^28^ Consequently, the suppression of electrostatic interactions enhances the relative selectivity of TDMC toward Ag(i) by emphasizing soft–soft interactions at the sulfur sites.

### 3.5. Desorption

**Fig. 5:**
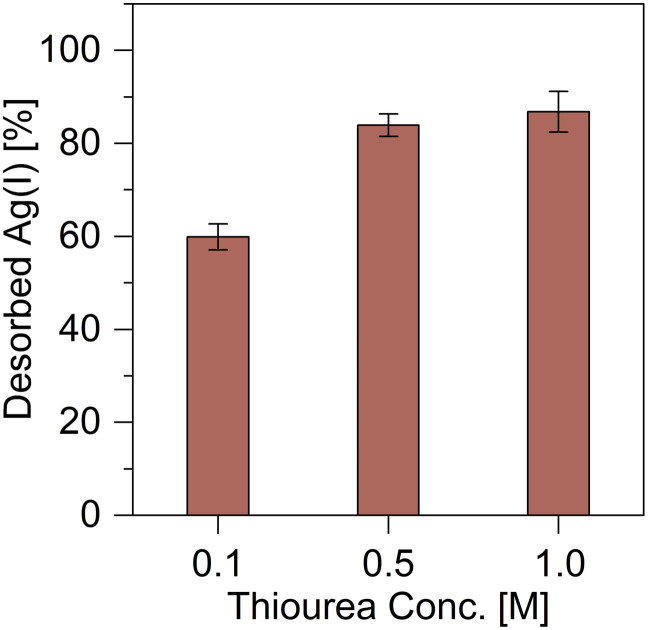
Effects of thiourea concentrations on Ag desorption.

The silver desorption performance of TDMC was investigated using a 0.1 M HNO_3_ solution. The desorption rate was 0.0%, thus indicating that no silver was desorbed (Table S1). Therefore, an aqueous solution of thiourea—a complexing agent—was employed. When desorption was performed with a 1.0 M thiourea solution, the desorption rate improved to 73.9% (Table S1). To further improve the desorption efficiency, 5.0 mL of 0.1 M HNO_3_ solutions containing 0.1, 0.5, and 1.0 M thiourea were evaluated (Fig. 5). Silver desorption efficiencies of 83.9% and 86.8% were achieved when using 0.5 M and 1.0 M thiourea solutions, respectively, thus indicating that the desorption reached a plateau at concentrations above 0.5 M. The limited improvement with increasing thiourea concentration suggests that a portion of silver remains in a strongly bound state or in locations inaccessible to the eluent.

### 3.6. Adsorption mechanism

**Fig. 6:**
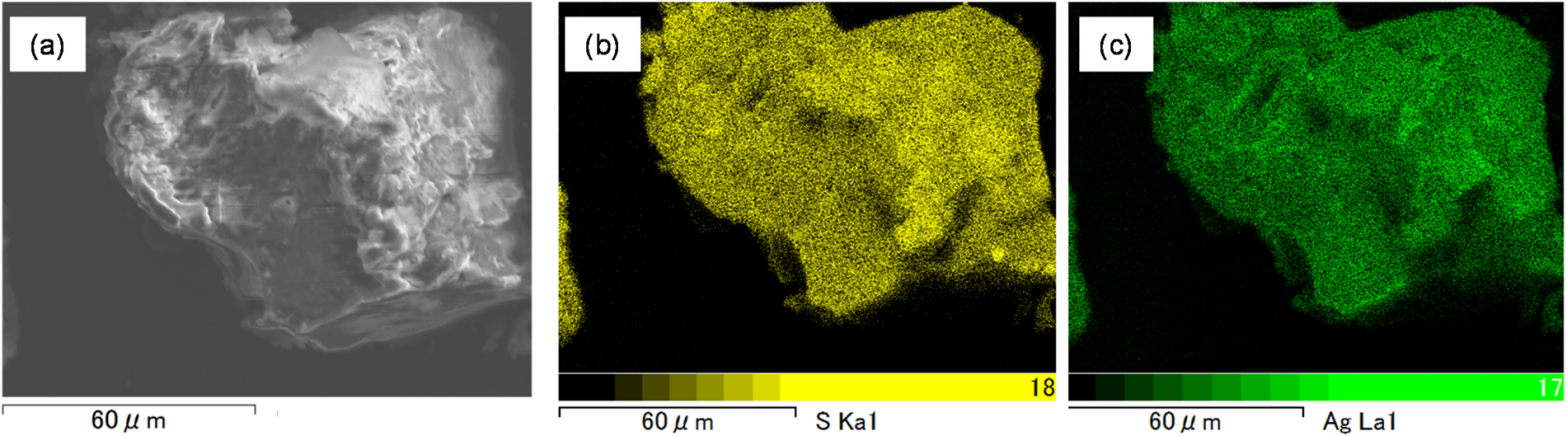
SEM image of Ag–TDMC (a) and EDX elemental mapping of (b) S and (c) Ag.

**Fig. 7:**
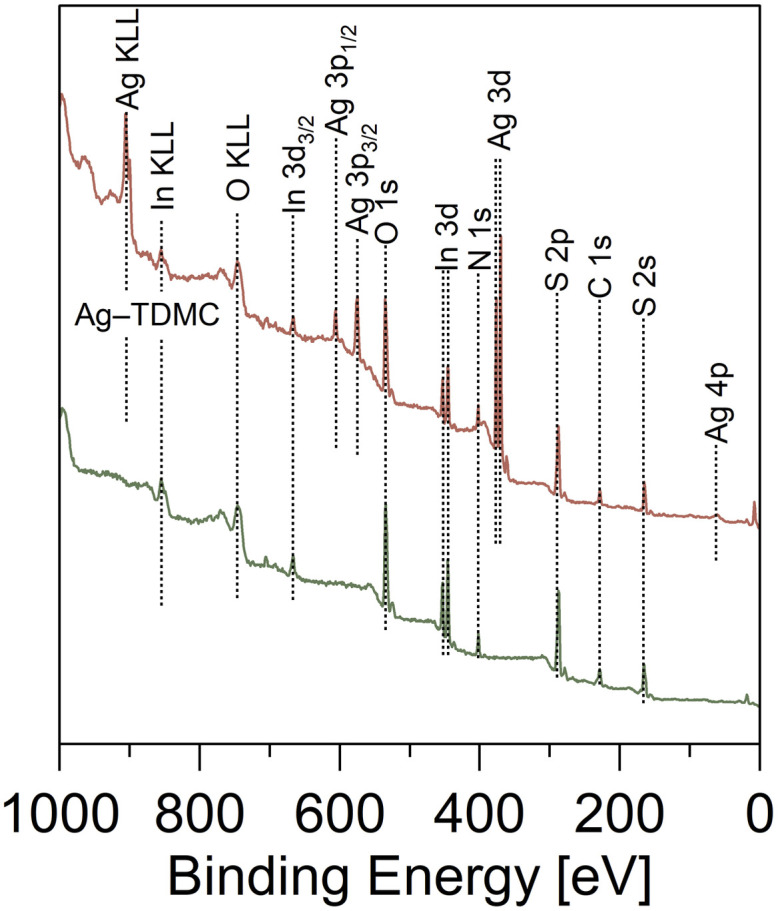
XPS wide-scan spectra.

SEM/EDX analyses were performed to examine the elemental distribution of the adsorbents. EDX elemental mapping revealed that sulfur and nitrogen atoms were distributed homogeneously over the surface of TDMC, thus indicating a uniform elemental distribution (Fig. 6). In the case of Ag–TDMC, Ag signals were clearly detected and uniformly distributed on the surface, thereby confirming that Ag adsorption occurred on TDMC. XPS wide-scan spectra were recorded for TDMC and Ag–TDMC to compare their surface chemical compositions. In the Ag–TDMC spectrum, Ag-derived peaks were observed, which were absent in pristine TDMC, whereas no other significant differences in elemental composition were detected (Fig. 7). Additionally, the Ag–TDMC spectrum exhibited characteristic Ag-related peaks at approximately 61, 370, 376, 575, and 906 eV. These results, together with the SEM/EDX analyses, indicate that Ag(i) adsorption occurred on the surface of TDMC.

**Fig. 8:**
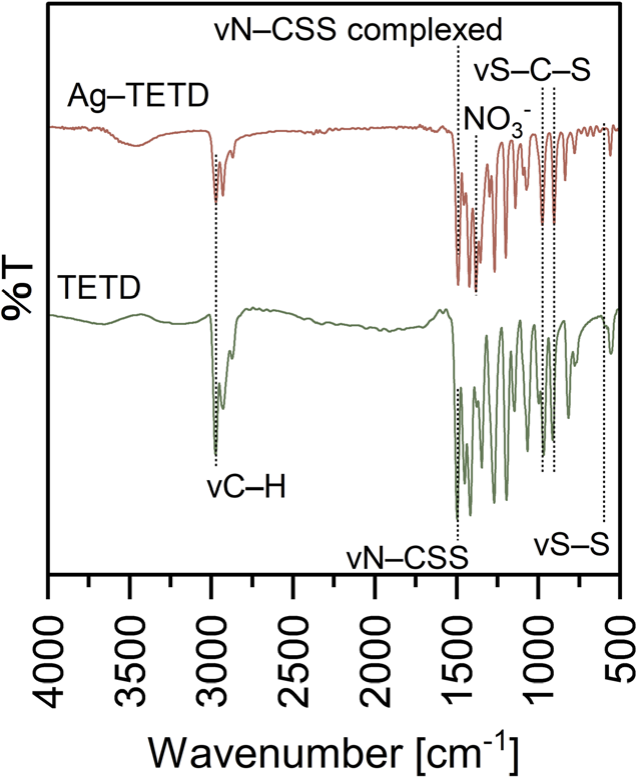
FT-IR spectrum of TDMC and Ag–TDMC.

FT-IR analysis was conducted to elucidate the functional groups involved in Ag(i) adsorption. The IR spectra of TDMC and Ag–TDMC are shown in Fig. 8. In the Ag–TDMC spectrum, the S–S stretching band at 615 cm^−1^, which was clearly observed for TDMC, disappeared, thus indicating the cleavage of the thiuram disulfide linkage during interaction with Ag(i). The C=S stretching vibration of the CS_2_ moiety (1059.7 cm^−1^) remained almost unchanged after Ag(i) adsorption, whereas the C–S band shifted to a lower wavenumber from 962.3 to 937.2 cm^−1^. This spectral pattern—minimal shift of the C=S band accompanied by a pronounced shift in the C–S region—is consistent with previous reports regarding on Ag–dithiocarbamate systems in which sulfur sites participate in Ag(i) binding.^36^ A strong band at 1391.4 cm^−1^ was observed in the Ag–TDMC spectrum, which is attributed to NO_3_^−^ derived from HNO_3_, thus implying that some nitrate remains associated with the adsorbent surface.

Collectively, these features indicate that the thiuram disulfide moiety undergoes S–S bond cleavage to generate a dithiocarbamate-like sulfur site, which then interacts with Ag(i) predominantly through the C–S sulfur atom. The slight shift of the N–CSS band to lower wavenumber suggests a redistribution of electron density within the N–CSS fragment, which is consistent with the increased electron delocalization upon Ag(i) interaction. Because the dithiocarbamate site formed after S–S bond cleavage is anionic, the Ag(i) associated with these sulfur donors would forms neutral or anionic surface species. Based on the results obtained from the FT-IR spectra, we can assume that the adsorption of Ag(i) converts thiuram disulfide into dithocarbamates, and that the produced dithiocarbamates interact with Ag(i) through the sulfur atoms of C–S bonds, thus yielding neutral or anionic surface species.

**Fig. 9:**
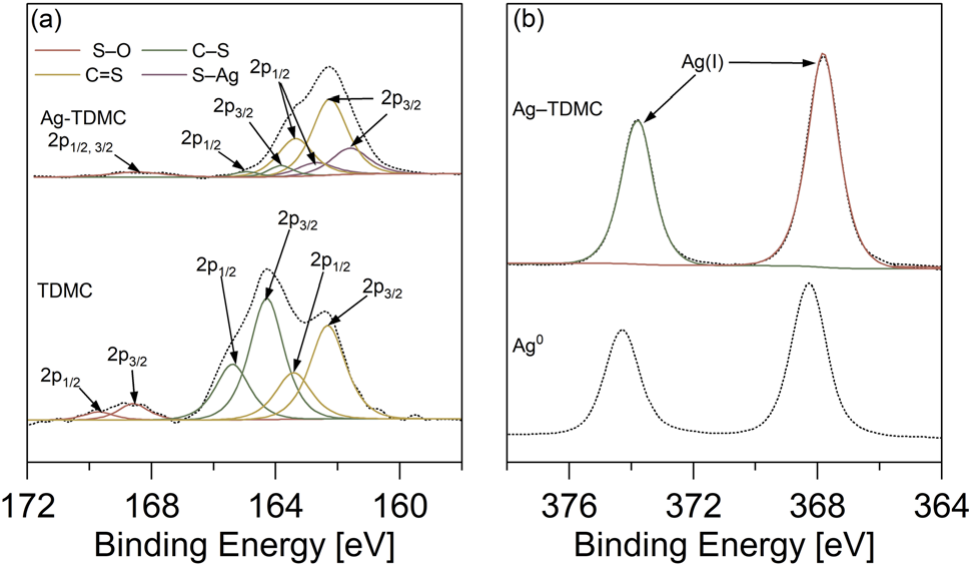
XPS (a) S 2p, (b) Ag 3d for TDMC and Ag–TDMC.

Narrow-scan XPS analysis was conducted to determine the ligand environment and oxidation state of Ag. To clarify the coordination states of sulfur in Ag–TDMC, S 2p narrow-scan spectra of the adsorbent before and after Ag(i) adsorption were obtained (Fig. 9). In TDMC, six peaks at 162.3, 163.4, 164.3, 165.4, 168.6 and 169.7 eV were demonstrated to describe the obtained experimental spectrum well. The first two peaks are attributed to C=S, the middle two correspond to C–S/S–S, and the last two represent S–O.^37,38^ After Ag adsorption, the spectrum changed significantly: two new peaks appeared at 161.1 and 162.7 eV, which are consistent with S–Ag bonding.^37–39^ These two peaks indicate that the adsorption of Ag(i) occurs through bonding between the sulfur atoms and Ag(i). Furthermore, the pronounced decrease in the peak intensity of the C–S bond suggests that Ag coordination occurs at the sulfur site of the C–S bond. Additionally, the peaks attributed to S–O bonds exhibited broadening after Ag(i) adsorption. This broadening suggests the possible formation of sulfone or sulfinic acid species–distinct from sulfate–during the adsorption process.^40,41^

To clarify the oxidation state of Ag in Ag–TDMC, Ag 3d narrow-scans were performed for Ag–TDMC and metallic Ag as a reference (Fig. 9). For metallic Ag, two peaks were observed at 368.3, and 374.3 eV, which represented Ag(0). In Ag–TDMC, two peaks were detected at 367.8 and 373.8 eV. Compared with the peaks for metallic Ag, those two peaks were located at slightly lower energy level. This suggests that the Ag in Ag–TDMC exists in a more oxidized form than Ag(0), which is supported by a previous report.^42^ Considering that NO_3_^−^ is the only potential oxidizing species present, coupled with the fact that it is does not oxidize Ag(i), the oxidation state of Ag is most likely +1. Therefore, the detected two peaks for Ag–TDMC can be assigned to Ag(i).

**Fig. 10:**
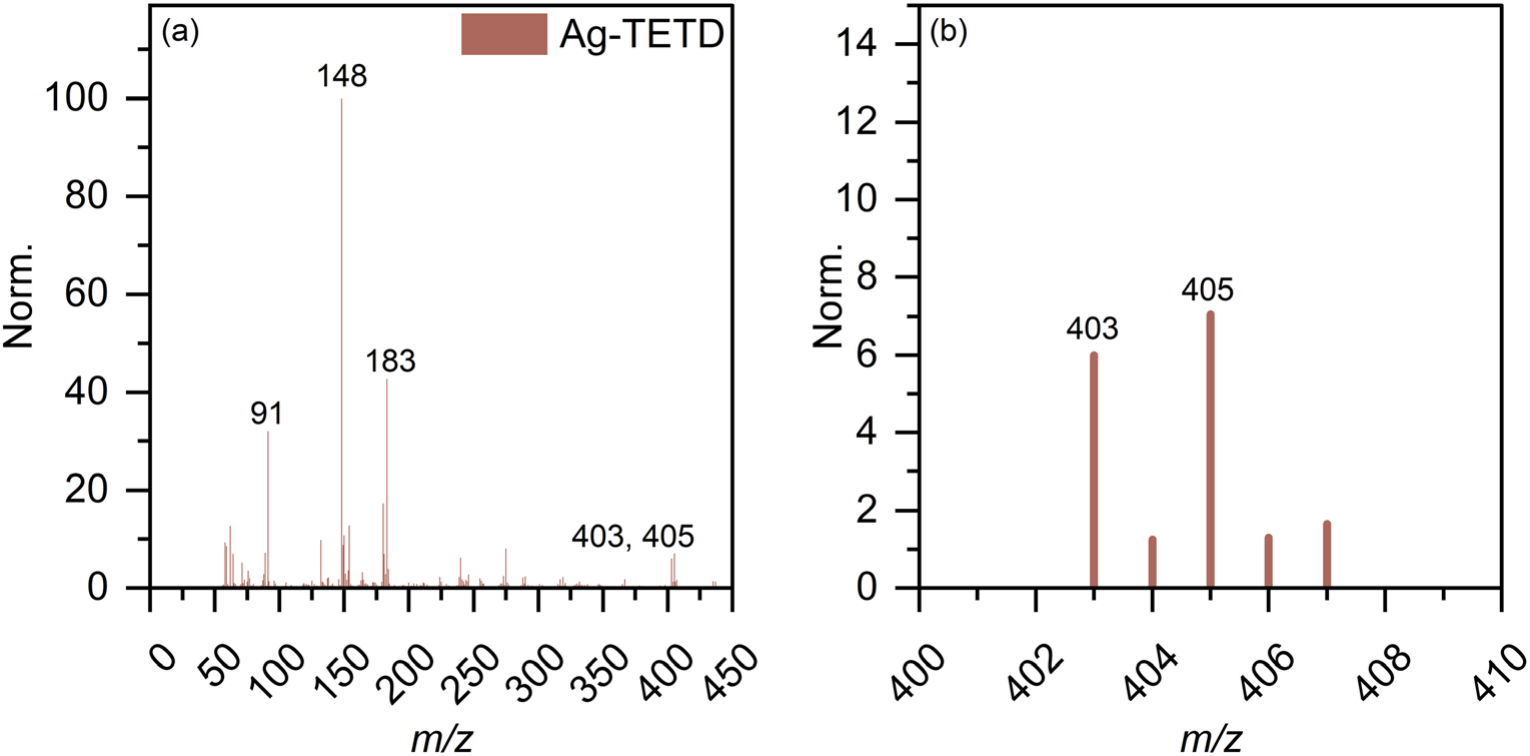
FAB(−)-MS spectrum (a) full range and (b) expanded view around *m*/*z* = 403 of Ag–TETD.

The spectroscopic investigations identified the functional groups involved in complexation and the oxidation state of Ag. However, the overall structure of the complex remains unclear. Mass spectrometry (MS) is an effective technique for structural analysis, however, its application for TDMC is challenging as TDMC is a high-molecular-weight polymer that cannot be easily ionized or detected using MS. Hence, we synthesized a low-molecular-weight analogue of Ag–TDMC, *i.e.*, Ag(i)-tetraethyl thiuram disulfide (TETD), for MS analysis. This analogue was selected as its IR and XPS spectra closely resemble those of Ag–TDMC (Fig. S2 and S3), thereby suggesting a similar coordination environment. In the positive-ion mode, the spectrum is dominated by fragment ions of TETD together with adduct ions arising from the gas-phase association of Ag^+^ (Fig. S4 and Table S2). Peaks at *m*/*z* 403 and 405 match the combined masses of ^107^Ag/^109^Ag and TETD, which is attributable to the adducts formed by the attachment of Ag^+^ to TETD. This interpretation is reasonable because the FT-IR results indicate the cleavage of the S–S bond and the subsequent formation of dithiocarbamate groups, thus suggesting that cationic solution-phase complexes are unlikely to form under our conditions (Fig. S2). By contrast, the anion-mode FAB-MS spectrum showed distinct signals at *m*/*z* 403 and 405 (Fig. 10). The 2u spacing and the intensity ratio are consistent with the natural isotopic envelope of ^107^Ag/^109^Ag, thereby supporting the presence of Ag-containing species with an overall Ag : TETD stoichiometry of 1 : 1. Therefore, the peaks at *m*/*z* 403 and 405 can be reasonably attributed to the isotopic components of the Ag–TETD complex (Table 3).

**Table 3:** FAB(−)–MS assign of Au(i)–TETD

No.	Observed *m*/*z*	Identified formula	Identified species	Calcd. *m*/*z*
1	91	C_3_H_7_O_3_^−^	Matrix monomer	91.0
2	148	C_5_H_11_NS_2_^−^	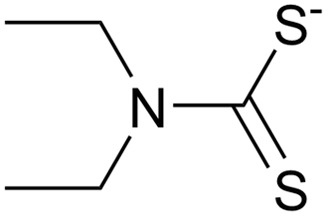	149.0
3	183	C_6_H_15_O_6_^−^	Matrix dimer	183.0
4	403	C_10_H_22_N_2_S_4_^107^Ag^−^	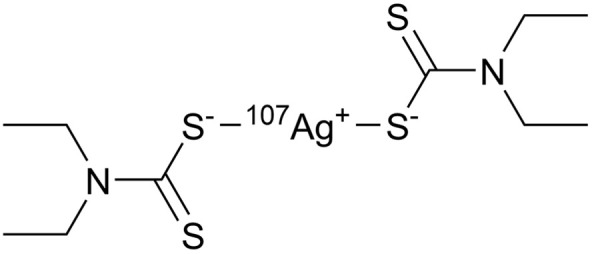	403.0
5	405	C_10_H_22_N_2_S_4_^109^Ag^−^	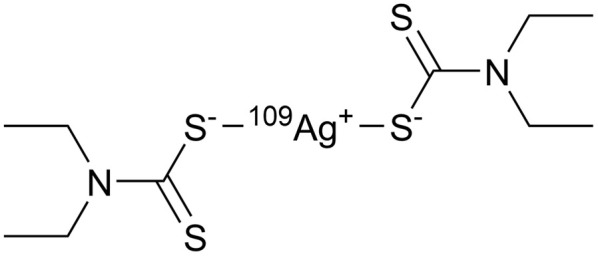	405.0

Based on FT-IR, XPS, and FAB-MS analyses, the adsorption of Ag(i) onto TDMC occurs *via* cleavage of thiuram–disulfide groups. In the presence of Ag(i), the soft Lewis acid property of Ag(i) can enable its initial coordination to the sulfur atoms (soft bases) of the thiuram disulfide moiety, thereby polarizing and activating the S–S bond.^43^ Since polarized disulfide bonds have been shown to undergo ionic cleavage in the presence of Lewis acids and bases,^43^ the coordination of Ag(i) is likely to induce a heterolytic S–S bond cleavage, thus generating an anionic sulfur (dithiocarbamate-like) and a cationic sulfur species. The changes observed in this study—S–S bond cleavage upon Ag(i) adsorption, coordination of Ag(i) to sulfur atoms, and the formation of S–O species—are consistent with this mechanism.

Unlike simple thiolates, where the high electron density on sulfur can fully neutralize the positive charge of Ag(i), the dithiocarbamate sulfur possesses a lower electron density due to resonance-induced charge delocalization over the N–C–S_2_ moiety. Consequently, the coordination of a single dithiocarbamate sulfur might not completely neutralize the cationic property of Ag(i), thus allowing the metal center to accept a second sulfur donor. This, combined with the preferred linear two-coordinate geometry of Ag(i) arising from its d^10^ electronic configuration, can promote the formation of a S–Ag–S structure bridging two dithiocarbamate units.

The excellent selectivity of TDMC toward Ag(i) can thus be attributed to the soft-acid property of Ag(i), which enables the initial coordination to the S–S bond site and subsequent activation of the chemisorption process (S–S bond activation → dithiocarbamate generation → metal complex formation). By contrast, borderline acids such as Pb(ii), Ni(ii), and Zn(ii) cannot coordinate effectively to the S–S bond due to their weak interaction with soft sulfur donors and thus fail to initiate this^43^ reaction-induced adsorption mechanism.

## 4. Conclusions

In this study, we investigated the applicability of a thiuram disulfide–anchored cellulose-based adsorbent, for the selective adsorption of Ag(i). The adsorbent maintained high selectivity for Ag(i) even in the presence of competing metal ions at concentrations up to 2000 times higher. Batch adsorption experiments revealed a maximum adsorption capacity of 3.6 mmol g^−1^. Spectroscopic analyses (FT-IR and XPS) and FAB-MS demonstrated that Ag(i) adsorption involved the cleavage of the S–S bond within the thiuram disulfide moiety, whereby the resulting two dithiocarbamate groups coordinated to Ag^+^ as monodentate ligands. Thus, reusing the adsorbent is likely to be challenging because of this adsorption mechanism.

## Author contributions

Shunsuke Taka: data curation, formal analysis, investigation, methodology, validation, visualization, writing – original draft, writing – review & editing. Keisuke Nakakubo: conceptualization, formal analysis, investigation, writing – original draft, writing – review & editing. Yuma Ito: investigation, writing – review & editing. Tsuyoshi Taniguchi: resources, writing – review & editing. Masaru Endo: resources. Kuo H. Wong: funding acquisition, writing – review & editing. Asami S. Mashio: funding acquisition, writing – review & editing. Tatsuya Nishimura: funding acquisition, resources, writing – review & editing. Katsuhiro Maeda: funding acquisition, resources, writing – review & editing. Hiroshi Hasegawa: conceptualization, funding acquisition, project administration, supervision, writing – review & editing.

## Conflicts of interest

Keisuke Nakakubo has patent #PCT/JP2022/034936 pending to License.

## Supplementary Material

RA-016-D5RA09818A-s001

## Data Availability

The data supporting the findings of this study are available in the supplementary information (SI) of this article. Supplementary information: additional experimental details and supporting data, including isotherm fitting information, synthesis of the Ag(I)-TETD reference complex, and supplementary FT-IR/XPS/FAB-MS results. See DOI: https://doi.org/10.1039/d5ra09818a.
