# Supplementary material for: Selective recovery of silver by thiuram disulfide-modified cellulose
Source: RSC Adv. 2026 Feb 24;16(12):10895–903. doi: 10.1039/d5ra09818a (PMC12930152; doi:10.1039/d5ra09818a)
Supplement: RA-016-D5RA09818A-s001 [file RA-016-D5RA09818A-s001.pdf]

Supplementary Information (SI) for RSC Advances.
This journal is © The Royal Society of Chemistry 2026

1. Isotherm studies

The experimental results were fitted using the Langmuir and Freundlich isotherm models. The non–linear and linear Langmuir equations are expressed as follows:

$$q_e = \frac{q_m K_L C_e}{(1 + K_L C_e)} \#(S1)$$

$$\frac{C_e}{q_e} = \frac{1}{q_m K_L} + \frac{C_e}{q_m} \#(S2)$$

where q_m (mol g^{-1}) is the theoretical maximum adsorption capacity, and K_L (L mol^{-1}) is the Langmuir constant. The nonlinear and linear Freundlich equations are as follows:

$$q_e = K_F C_e^{1/n} \#(S3)$$

$$\log q_e = \log K_F + \frac{1}{n} \log C_e \#(S4)$$

where K_F represents the Freundlich constant, and n refers to the adsorption intensity.

2. Synthesis ligands and complex

2.1. Ag(I)–TETD

To a solution of $AgNO_3$ (68.0 mg, 0.4 mmol) in 0.1 M HNO_3 solution (100 mL) was added to TETD in an Erlenmeyer flask. After an hour of stirring, the resulting precipitate was collected by filtration, washed with water to remove residual silver ions, and dried overnight in a thermostatic chamber at 40°C, yielding an orange solid. Since its XPS spectrum suggests that Ag exists in the +1 oxidation state, the obtained compound was named Ag(I)–TETD.

3. Supplementary Figures & Tables

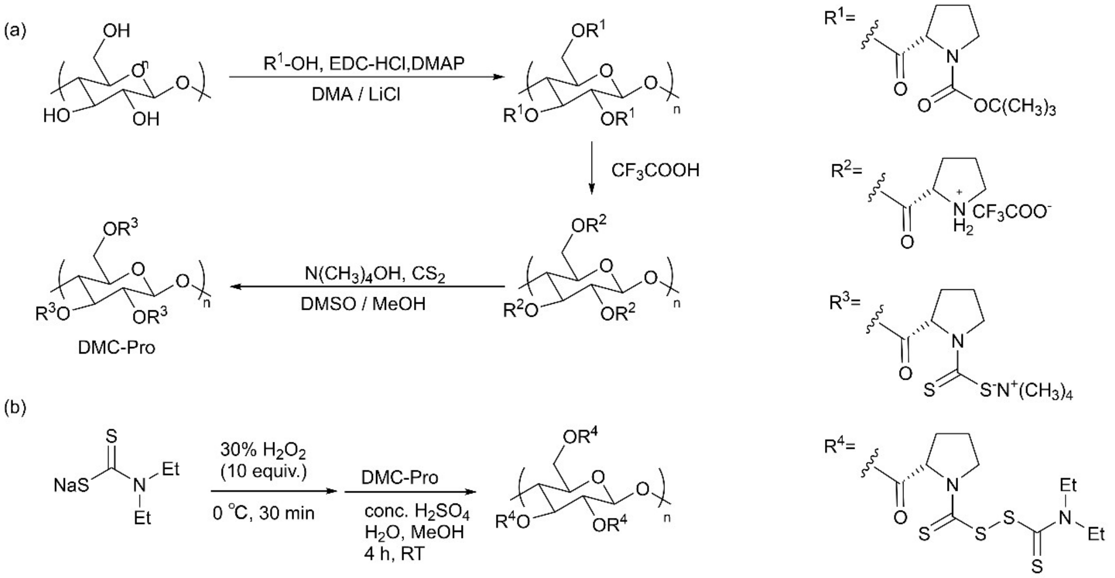

Fig. S1. TDMC synthesis protocol

Table S1. Effects of solution composition on Ag desorption.

Solution	Desorbed Ag(I) [%]
0.1 M HNO_3	0.0±0.0
1.0 M Thiourea	75.5±5.0

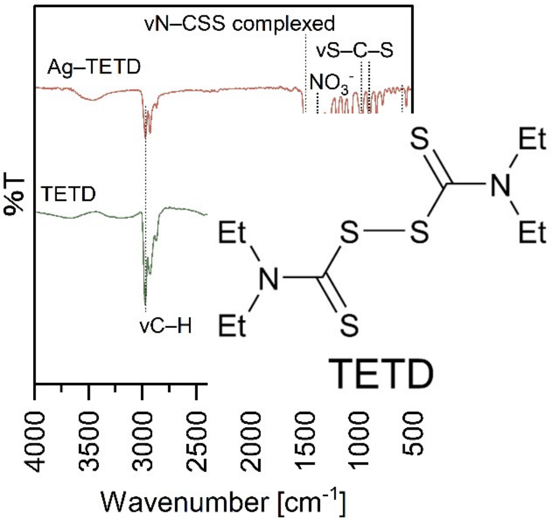

Fig. S2. FT–IR spectrum of TETD, Ag–TETD

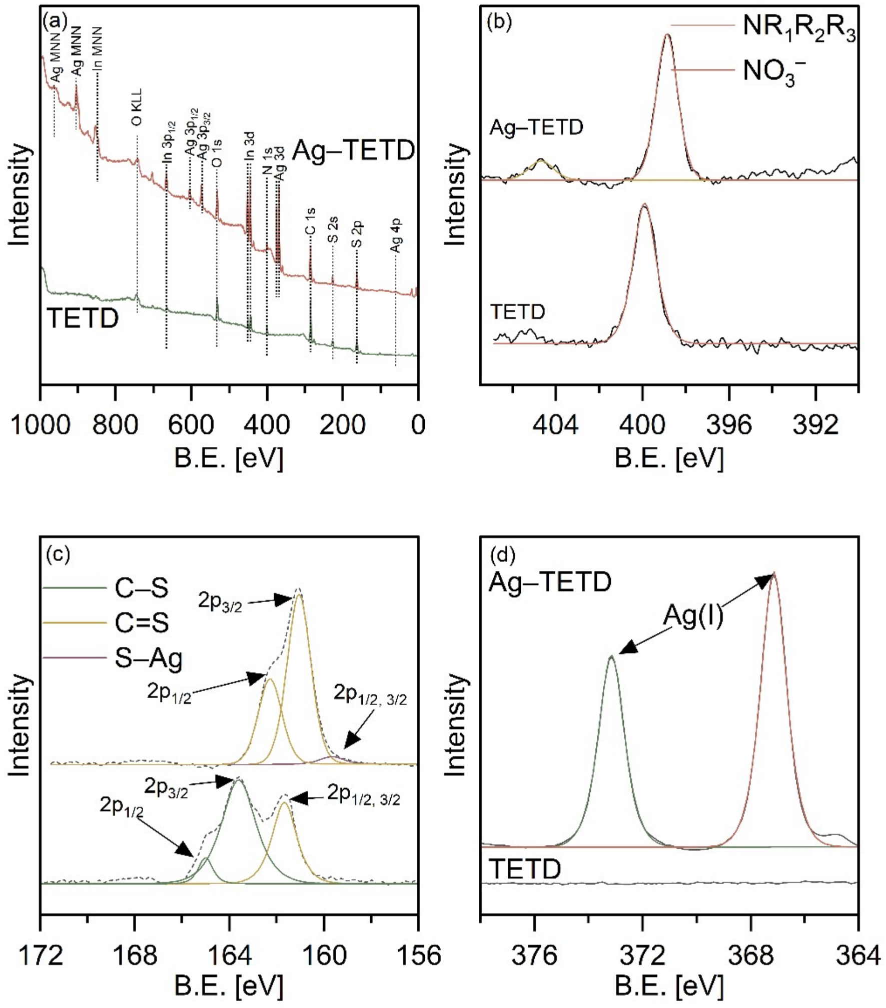

Fig. S3. XPS (a) wide-scan spectra and narrow-scan spectra of (b) N 1s, (c) S 2p (d) Ag 3d for TETD and Ag–TETD

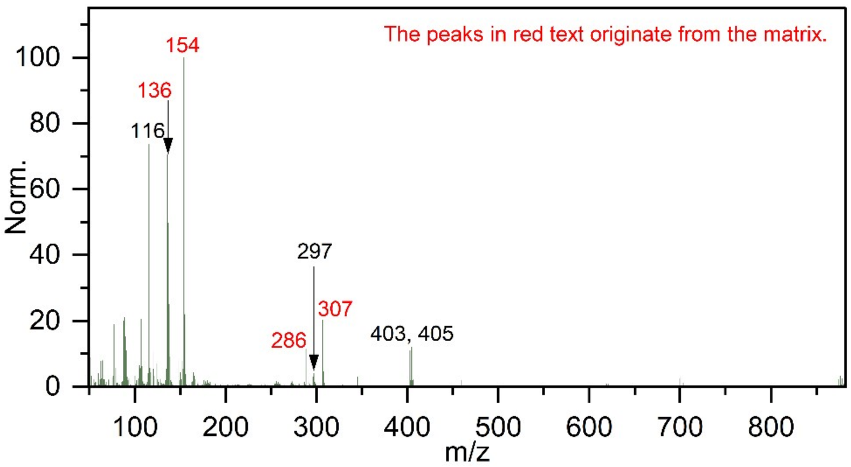

Fig. S4. FAB(+)-MS spectrum of Ag–TETD

Table S2. FAB(+)–MS assignment for Au(I)–TETD

No.	Observed *m/z*	Identified formula	Identified species	Calcd. *m/z*
1	116	$C_5H_{10}NS^+$	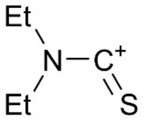	116.0
2	136	$C_7H_6NO_2^+$	Matrix fragment	136.0
3	136	$[C_7H_7NO_3 + H]^+$	Matrix monomer	153.0
4	286	$[C_{14}H_{13}N_2O_5 + H]^+$	Matrix dimer fragment	286.0
5	297	$[C_{10}H_{20}N_2S_4 + H]^+$	TETD	297.1
6	307	$[C_{14}H_{14}N_2O_6 + H]^+$	Matrix dimer	307.0
7, 8	403, 405	$C_{10}H_{22}N_2S_4$ + $^{107}Ag^+$ or $^{109}Ag^+$	—	403.0<br>407.0

Reference

[1] H.J.A. Blaauw, R.J.F. Nivard, G.J.M. Van Der Kerk, Chemistry of organogold compounds, J Organomet Chem 2 (1964) 236–244. https://doi.org/10.1016/S0022-328X(00)80517-3.

[2] B.P. Block, J.C. Bailar, The reaction of gold(III) with some bidentate coordinating groups, Journal of American Chemical Society 73 (1951) 4722–4725.

[3] D. Coucouvanis, The chemistry of the dithioacid and 1,1-dithiolate complexes, in: S.J. Lippard (Ed.), Progress in Inorganic Chemistry, Interscience Publishers, 1970: pp. 233–371.
